# Optimizing Artificial Intelligence Thresholds for Mammographic Lesion Detection: A Retrospective Study on Diagnostic Performance and Radiologist–Artificial Intelligence Discordance

**DOI:** 10.3390/diagnostics15111368

**Published:** 2025-05-29

**Authors:** Taesun Han, Hyesun Yun, Young Keun Sur, Heeboong Park

**Affiliations:** 1 Department of Radiology, Park Heeboong Surgical Clinic, 203, Geowerk, 7, Hyowon-ro, 256beon-gil, Gwonseon-gu, Suwon-si 16571, Gyeonggi-do, Republic of Korea; 2 Department of Family Medicine, Park Heeboong Surgical Clinic, 203, Geowerk, 7, Hyowon-ro, 256beon-gil, Gwonseon-gu, Suwon-si 16571, Gyeonggi-do, Republic of Korea; hssun0804@gmail.com; 3 Department of Radiology, Dongsuwon General Hospital, 165, Jungbu-daero, Paldal-gu, Suwon-si 16494, Gyeonggi-do, Republic of Korea; youngkeunsur@gmail.com; 4 Department of General Surgery, Park Heeboong Surgical Clinic, 203, Geowerk, 7, Hyowon-ro, 256beon-gil, Gwonseon-gu, Suwon-si 16571, Gyeonggi-do, Republic of Korea; heeboong@gmail.com

**Keywords:** artificial intelligence, mammography, breast cancer, BI-RADS, diagnostic thresholds, dense breast, radiologist–AI discordance, computer-aided diagnosis

## Abstract

**Background/Objectives:** Artificial intelligence (AI)-based systems are increasingly being used to assist radiologists in detecting breast cancer on mammograms. However, applying fixed AI score thresholds across diverse lesion types may compromise diagnostic performance, especially in women with dense breasts. This study aimed to determine optimal category-specific AI thresholds and to analyze discrepancies between AI predictions and radiologist assessments, particularly for BI-RADS 4A versus 4B/4C lesions. **Methods**: We retrospectively analyzed 194 mammograms (76 BI-RADS 4A and 118 BI-RADS 4B/4C) using FDA-approved AI software. Lesion characteristics, breast density, AI scores, and pathology results were collected. A receiver operating characteristic (ROC) analysis was conducted to determine the optimal thresholds via Youden’s index. Discrepancy analysis focused on BI-RADS 4A lesions with AI scores of ≥35 and BI-RADS 4B/4C lesions with AI scores of <35. **Results**: AI scores were significantly higher in malignant versus benign cases (72.1 vs. 20.9; *p* < 0.001). The optimal AI threshold was 19 for BI-RADS 4A (AUC = 0.685) and 63 for BI-RADS 4B/4C (AUC = 0.908). In discordant cases, BI-RADS 4A lesions with scores of ≥35 had a malignancy rate of 43.8%, while BI-RADS 4B/4C lesions with scores of <35 had a malignancy rate of 19.5%. **Conclusions**: Using category-specific AI thresholds improves diagnostic accuracy and supports radiologist decision-making. However, limitations persist in BI-RADS 4A cases with overlapping scores, reinforcing the need for radiologist oversight and tailored AI integration strategies in clinical practice.

## 1. Introduction

### 1.1. Background

Breast cancer is the most commonly diagnosed cancer among women worldwide, with an estimated 2.3 million new cases and 670,000 deaths in 2022, highlighting the global importance of early detection and screening programs in enhancing treatment outcomes and survival rates [1,2]. Mammographic screening programs have significantly increased the detection of breast cancer, yet they have also led to a rise in screening volume, placing a substantial burden on radiologists and contributing to variability in interpretation and diagnostic fatigue [3].

To address these challenges, artificial intelligence (AI)-based computer-aided detection/diagnosis (CADe/x) systems have been developed to assist radiologists by enhancing diagnostic accuracy and efficiency [4,5,6,7]. Recent advancements in deep learning have enabled AI to achieve detection performance comparable to, or in some cases surpassing, that of radiologists [8,9,10,11]. AI-based mammography tools commonly assign abnormality scores to quantify malignancy risk, using either a 1–10 or 0–100 scale [12,13].

However, the effectiveness of AI depends on the score threshold chosen to classify a lesion as suspicious. Most AI systems generate a continuous abnormality score to represent the likelihood of malignancy. This score is calculated based on image features such as margins, density, and microcalcification patterns. The interpretation of this score requires context-dependent thresholds to guide recall or biopsy decisions. Lower thresholds prioritize sensitivity but increase false positives, whereas higher thresholds reduce workload but may miss cancers [14].

A key limitation of current AI applications is that a single threshold is often applied across all lesion categories, despite their differing malignancy risks. The Breast Imaging Reporting and Data System (BI-RADS) classification system, developed by the American College of Radiology, categorizes suspicious breast lesions into subtypes 4A, 4B, and 4C, with increasing probabilities of malignancy: 4A (>2–10%), 4B (>10–50%), and 4C (>50–<95%) [15]. These subcategories guide clinical decisions ranging from follow-up imaging to biopsy.

In clinical practice, BI-RADS 4A lesions—which carry a relatively low suspicion for malignancy—are generally recommended for biopsy according to ACR BI-RADS guidelines. However, in select cases with low-risk features or patient-specific considerations, short-term follow-up imaging may also be considered [15]. Applying a uniform threshold across these categories may lead to unnecessary recalls or, conversely, missed malignancies.

### 1.2. Literature Review

AI has shown particular promise in detecting cancers in women with dense breasts, where conventional mammography has limited sensitivity. Koch et al. reported that AI achieved 80.9% sensitivity in high-density breasts, outperforming radiologists’ 60.6–62.8% sensitivity in the same population [16]. Kwon et al. reported that AI-based software outperformed radiologists in recall rate, specificity, PPV, and AUC, with disparities being most prominent in extremely dense breasts [17].

These findings suggest that AI systems can provide substantial diagnostic value, but their performance significantly depends on how their outputs—particularly abnormality scores—are interpreted.

Studies have demonstrated the need for optimizing AI thresholds; for instance, Dahlblom et al. reported that a threshold of 62 yielded 75% sensitivity and 90.8% specificity for digital mammography, while Kizildag Yirgin et al. applied a 34.5% threshold, achieving 72.8% sensitivity, 88.3% specificity, and a 72.7% cancer detection rate [18,19]. Similarly, Seker et al. (2024), reported an optimal threshold of 30.4 with 72.4% sensitivity and 92.9% specificity, achieving an AUC of 89.6% [20]. These findings highlight the clinical necessity of optimizing AI thresholds to balance sensitivity, specificity, and recall performance (Table 1).

### 1.3. Study Rationale and Objectives

Despite significant advances in AI-based mammographic analysis, most previous studies have applied a single score threshold across all lesion types, overlooking the variation in malignancy risk among BI-RADS subcategories. Moreover, few have directly examined radiologist–AI discordance in borderline categories such as BI-RADS 4A versus 4B/4C.

To address these gaps, this study aims to achieve the following contributions:

- Establish BI-RADS-specific AI thresholds using both Youden-optimal and sensitivity-prioritized criteria, enabling tailored clinical applications.
- Explore clinical performance trade-offs across threshold values to support optimal decision-making in real-world settings.
- Analyze radiologist–AI discordance in BI-RADS 4A and 4B/4C subgroups to identify scenarios in which AI may diverge from human judgment.

Currently, AI is primarily used as a secondary reader in clinical practice rather than as a standalone tool, assisting radiologists in decision-making [21]. Therefore, establishing case-specific AI thresholds that can support radiologists’ decisions would be beneficial. By refining score interpretation and identifying threshold-driven mismatch patterns, we seek to improve clinical decision-making and optimize AI integration into radiology workflows.

## 2. Materials and Methods

This section details the retrospective study design, patient selection criteria, mammographic imaging protocol, AI scoring procedure, radiologist assessment, and statistical analyses used to evaluate diagnostic performance, category-specific threshold optimization, and radiologist–AI discordance.

### 2.1. Study Design and Ethical Considerations

This retrospective study was conducted at a single center and involved a radiologist with over 10 years of experience in mammography interpretation. It was approved by the institutional review board, with a waiver of informed consent due to the retrospective nature of the study and the use of de-identified data.

### 2.2. Patient Selection and Data Collection

Mammographic cases performed between 21 June 2024 and 13 January 2025 were reviewed. A total of 194 female patients aged 28–83 years (mean age 49.5 ± 9.2 years) were included. Among them, 79.9% (155) had dense breast tissue classified as breast density 3 or 4. Only patients with mammographic lesions initially categorized as BI-RADS 4 (4A, 4B, or 4C) were included in this study. Cases with BI-RADS 1–3 or BI-RADS 5 assessments were excluded to focus the analysis on intermediate-risk lesions. All included cases included corresponding histopathological confirmation via core needle biopsy or surgical excision. Patients were categorized into two groups based on their initial BI-RADS classification: the BI-RADS 4A group (*n* = 76) and the BI-RADS 4B/4C group (*n* = 118).

This study focused on BI-RADS category 4 lesions, particularly 4A, as they represent the most challenging diagnostic group. We compared them with 4B/4C lesions to assess how AI thresholds can be optimized across varying malignancy risks. Lesions classified as BI-RADS 1–3 are generally managed with imaging follow-up, while BI-RADS 5 lesions are typically treated as malignant regardless of AI score. Therefore, the inclusion of category 4 lesions provides the most meaningful context for evaluating and calibrating AI thresholds.

### 2.3. Imaging Protocol and Interpretation

Mammography was performed using full-field digital mammography units (Selenia, Hologic Inc., Marlborough, MA, USA), with each patient undergoing standard bilateral craniocaudal (CC) and mediolateral oblique (MLO) views. The mammograms were retrospectively analyzed using Lunit INSIGHT MMG, version 1.1.9.2. (Lunit Inc., Seoul, Republic of Korea), an AI-powered computer-aided detection/diagnosis (CADe/x) tool designed to highlight suspicious lesions and assign abnormality scores ranging from 0 to 100. The AI model was developed using over 50,000 breast cancer cases and trained on more than 200,000 mammographic cases from Korea, the United States, and the United Kingdom. Thus, it received regulatory approval from the U.S. Food and Drug Administration (FDA) and the Korean Ministry of Food and Drug Safety (MFDS) [8].

The AI abnormality score reflects the model’s estimated likelihood of malignancy based on image features such as mass shape, margin, and microcalcification patterns. In clinical settings, this score is often used as an additional reference to support radiologist decision-making, particularly in screening environments where early malignancy detection is critical.

AI analysis was conducted independently, without reference to radiologist assessments. Lesions located at the periphery of the image, within the skin, or in areas where more than 50% of the lesion was cropped were excluded to minimize false positive results.

As this study aimed to evaluate the diagnostic performance of a pre-trained AI model, no dataset partitioning into training or validation sets was performed. All cases were used as an independent clinical validation cohort. As Lunit INSIGHT MMG is a proprietary and commercially deployed system, detailed hyperparameter settings are not accessible to users and were not modified for the purposes of this study.

### 2.4. Radiologist Review and Comparative Analysis

Radiologists initially assigned BI-RADS scores using the fifth edition of Bi-RADS for Mammography and Ultrasound without AI assistance [15]. Key recorded variables included the AI score, patient age, initial BI-RADS classifications, breast density (BI-RADS 1–4), lesion characteristics (margin, density, microcalcifications, or parenchymal distortion), radiologist recall decisions, biopsy results, malignancy status, and ultrasound (US) visibility.

### 2.5. Discordance Analysis

A discordance analysis was performed to evaluate the differences between the AI predictions and the radiologist assessments, focusing on cases in which AI classifications diverged from human interpretation. This involved a detailed review of imaging findings, lesion characteristics, and final pathology results to identify patterns in AI-detected versus radiologist-detected abnormalities. Insights from this analysis aimed to refine AI algorithms and enhance their accuracy in clinical settings.

### 2.6. Statistical Analysis

Statistical analyses were conducted to evaluate the diagnostic performance of the AI system. A receiver operating characteristic (ROC) curve analysis was performed using DeLong’s method to determine the area under the curve (AUC) and corresponding 95% confidence intervals (CIs) for each BI-RADS group [22]. Sensitivity, specificity, and optimal thresholds for the AI scores were derived using Youden’s index [23].

Mean age differences between the BI-RADS 4A and 4B/4C groups were assessed using an independent *t*-test. The proportion of dense breasts (categories 3 and 4) was compared between the two groups using a chi-square test. Malignancy rates were then compared between these groups using the Z-test for proportions. Mean AI scores were assessed between these groups using Welch’s *t*-test.

An independent *t*-test was performed to compare the AI scores between benign and malignant cases. Lesion characteristics, including microcalcification morphology (punctate, amorphous, fine pleomorphic, and fine linear/branching) and mass margins (circumscribed, irregular, and spiculated), were evaluated in relation to the AI scores. Microcalcification morphology and AI score differences were analyzed using one-way ANOVA. Mass margin differences were also assessed using one-way ANOVA, while mass density, architectural distortion, and other binary comparisons were analyzed using independent *t*-tests.

For cases with discrepancies between AI and radiologists, subgroup analyses were performed. Two subgroups were defined: BI-RADS 4A cases with AI scores of ≥35 and BI-RADS 4B/4C cases with AI scores of <35. The mean AI scores between malignant and benign cases within these subgroups were compared using independent *t*-tests.

These statistical approaches—including ROC analysis, Youden index optimization, and subgroup discordance evaluation—are widely used in diagnostic imaging studies and were selected for their proven efficiency and clinical relevance in evaluating AI performance [16,17,18,19,20].

All statistical tests were two-tailed, with a significance threshold of *p* < 0.05. Analyses were conducted using SPSS Version 26 (IBM Corp., Armonk, NY, USA) and R statistical software (Version 4.0.2; R Foundation for Statistical Computing, Vienna, Austria).

Graphs and ROC curves were generated using Python-based tools, including matplotlib (v3.7.1; matplotlib development team, open-source) and seaborn (v0.12.2; Michael Waskom and contributors, open-source). Python code was partially generated with the assistance of ChatGPT (GPT-4, OpenAI; version as of April 2025; OpenAI, San Francisco, CA, USA), which was solely used for code syntax generation. All statistical analyses, figure interpretations, and final decisions were performed independently by the authors.

**Figure 1:**
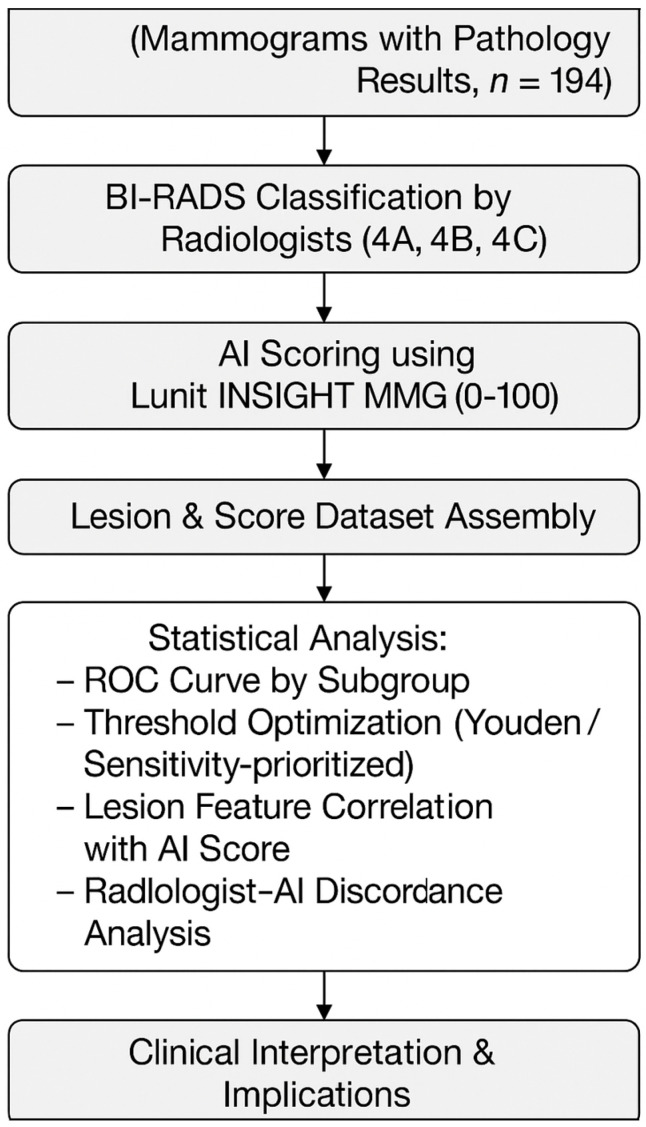
Workflow diagram of study design and analysis.

Figure 1 provides a visual summary of the retrospective study workflow.

## 3. Results

This section presents the comparative results of AI scores across BI-RADS subgroups, lesion characteristics, and malignancy status, followed by performance metrics and subgroup analyses for discordant cases.

### 3.1. Baseline Characteristics and Group Comparisons

The mean ages of patients in the BI-RADS 4A and 4B/4C groups were 49.3 ± 8.7 and 49.6 ± 9.4 years, respectively, with no significant difference observed. The proportion of dense breasts (categories 3 and 4) was 82.9% in BI-RADS 4A and 78.0% in BI-RADS 4B/4C, also showing no significant difference (*p* > 0.05).

Among the 194 analyzed cases, 88 (45.4%) were malignant, comprising 34 cases of ductal carcinoma in situ (DCIS), 51 cases of invasive ductal carcinoma (IDC), 1 case of lobular carcinoma in situ (LCIS), and 2 cases of mucinous carcinoma. Malignancy rates were significantly higher in BI-RADS 4B/4C lesions (*n* = 71, 60.2%) compared to BI-RADS 4A lesions (*n* = 17, 22.4%) (*p* < 0.001).

### 3.2. AI Score Distribution by Lesion Category

AI scores were significantly elevated in BI-RADS 4B/4C cases (58.1 ± 37.9) compared to BI-RADS 4A cases (22.4 ± 22.7) (*p* < 0.001). Malignant cases had higher AI scores (72.1 ± 32.7) than benign cases (20.9 ± 20.9) (*p* < 0.001).

### 3.3. Lesion Characteristics and AI Score Correlation

Lesion characteristics were further examined by BI-RADS category. BI-RADS 4A lesions most frequently exhibited grouped microcalcifications, particularly punctate (*n* = 7, 41.2%) and punctate/amorphous (*n* = 5, 29.4%) morphologies. In contrast, BI-RADS 4B/4C lesions predominantly showed segmental distribution (*n* = 23, 76.7%), with a variety of morphologies, including fine pleomorphic (*n* = 6, 20.0%), fine linear (*n* = 5, 16.7%), amorphous (*n* = 7, 23.3%), and coarse heterogeneous (*n* = 5, 16.7%) types. One-way ANOVA showed that fine pleomorphic and fine linear microcalcifications were associated with significantly higher AI scores (*p* < 0.001). Similarly, lesions with spiculated or irregular margins also showed significantly elevated scores (*p* < 0.001) (Table 2).

### 3.4. ROC Analysis and Threshold Optimization

**Figure 2:**
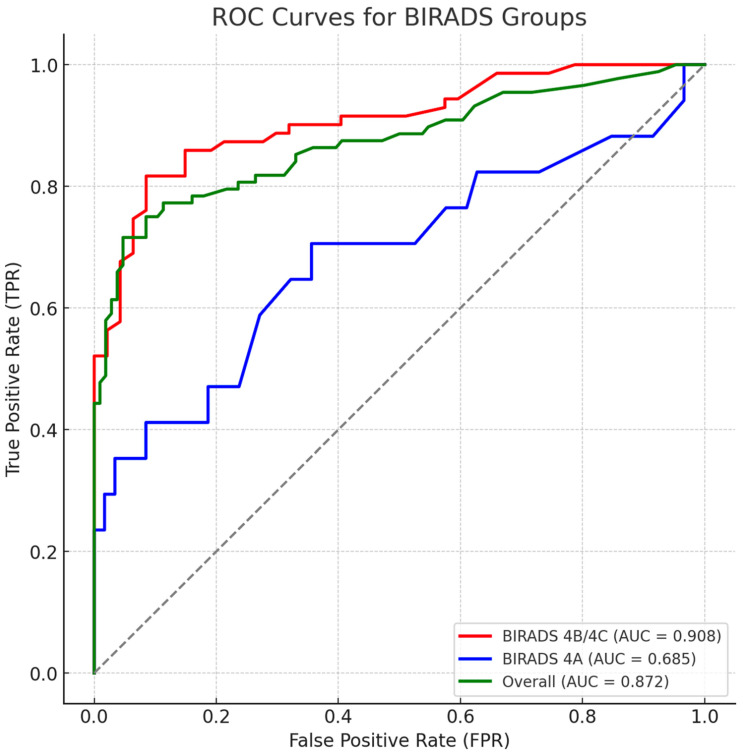
ROC curves of AI score performance by BI-RADS subgroup. Receiver operating characteristic (ROC) curves illustrate the diagnostic performance of the AI score in distinguishing malignant from benign lesions across BI-RADS subgroups. The area under the curve (AUC) was highest for the BI-RADS 4B/4C group (AUC = 0.908), followed by the overall group (AUC = 0.872), and lowest for the 4A group (AUC = 0.685). These curves reflect stronger predictive capabilities in higher-risk subgroups, supporting AI-assisted decision-making in biopsy recommendations. The dotted diagonal represents the line of no-discrimination (AUC = 0.5).

An ROC analysis was performed to assess the diagnostic performance of the AI model in differentiating malignant from benign lesions. The AUC for the BI-RADS 4A group was 0.685 (95% CI: 0.58–0.79), while the BI-RADS 4B/4C group achieved a higher AUC of 0.908 (95% CI: 0.76–1.00). The overall AUC was 0.872 (95% CI: 0.77–0.98), reflecting strong AI performance, particularly in detecting BI-RADS 4B/4C lesions (Figure 2).

Youden-optimal thresholds were 19 for BI-RADS 4A (sensitivity: 0.71, specificity: 0.64; AUC = 0.685), 63 for BI-RADS 4B/4C (sensitivity: 0.82, specificity: 0.92; AUC = 0.908), and 63 for the overall cohort (sensitivity: 0.72, specificity: 0.95; AUC = 0.872).

**Figure 3:**
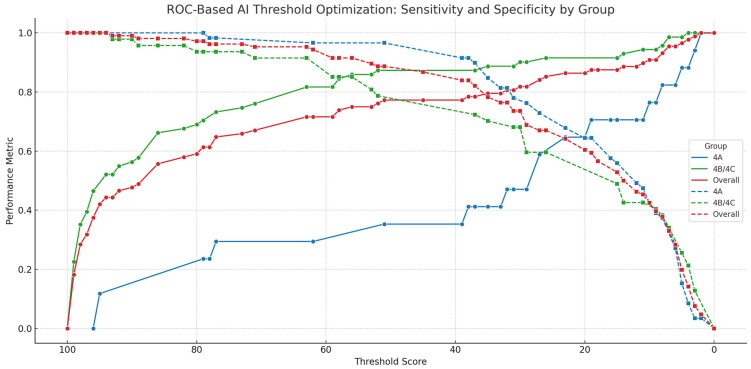
Sensitivity and specificity across threshold values by BI-RADS subgroup. Sensitivity and specificity trends of the AI score across different threshold values for the BI-RADS 4A, 4B/4C, and overall groups. The *x*-axis represents decreasing threshold values (right to left), and the *y*-axis represents performance metrics. Solid lines indicate sensitivity, and dashed lines indicate specificity. The chart highlights how lower thresholds result in higher sensitivity but reduced specificity, allowing clinicians to adjust cutoff points based on the desired clinical balance. This visualization supports both Youden-optimal and sensitivity-prioritized threshold selection strategies.

When sensitivity was prioritized for clinical application, thresholds were adjusted as follows: 8 for BI-RADS 4A (sensitivity: 0.82, specificity: 0.37), 58 for BI-RADS 4B/4C (sensitivity: 0.85, specificity: 0.85), and 35 for the overall group (sensitivity: 0.80, specificity: 0.78) (Figure 3, Table 3). These alternative cutoffs provide clinically adaptable options depending on the diagnostic objective.

### 3.5. Discordance Analysis Between AI and Radiologists

Subgroup analysis was conducted to explore diagnostic discordance between AI and radiologist assessments. Two groups were defined: (1) BI-RADS 4A cases with AI scores of ≥35, and (2) BI-RADS 4B/4C cases with AI scores of <35. A threshold of 35 was chosen to reflect high sensitivity (0.80) with acceptable specificity (0.78).

In the BI-RADS 4A subgroup, 16 cases had AI scores of ≥35, with 10 (62.5%) showing grouped microcalcifications without associated masses. Malignancy was confirmed in seven cases (DCIS = 3, IDC = 4), yielding a malignancy rate of 43.8%. The mean AI scores were significantly higher in malignant lesions (73.9, range 38–95) than in benign ones (45.2, range 35–78) (*p* = 0.013). Findings included focal asymmetry with spiculated margins, grouped punctate microcalcifications, and circumscribed or irregularly marginated masses.

In the BI-RADS 4B/4C group with AI scores of <35, 41 cases were identified (39 BI-RADS 4B, 2 BI-RADS 4C). Most lesions exhibited microcalcifications (*n* = 38, 92.7%), predominantly segmental (*n* = 34, 82.9%) or grouped (*n* = 4, 9.8%). Morphologies included amorphous (*n* = 15, 39.5%) and amorphous/punctate (*n* = 21, 55.3%). Eight malignant cases (19.5%) were confirmed, including six DCIS and two IDC. However, no significant difference in AI scores was found between benign (10.9, range 0–31) and malignant lesions (13.4, range 4–30) (*p* > 0.05).

## 4. Discussion

Recent deep learning-based models, such as RGGC-UNet, GSN-HVNet, and RTLinearFormer, have achieved strong performance in segmentation and classification tasks across medical imaging applications, particularly in pathology [24,25,26]. Although these models target different modalities, they exemplify the rapid evolution of AI technology in diagnostics. In this context, our study applies a threshold-based AI scoring system specifically tailored for mammographic lesion stratification, contributing to the expanding field of AI-assisted image interpretation.

This section discusses the clinical implications of our findings, particularly the importance of category-specific AI thresholds and radiologist–AI discordance, in optimizing breast cancer diagnostic workflows.

### 4.1. Interpretation of Diagnostic Thresholds and AI Performance

The motivation for this study stemmed from the observation that AI-assisted mammography analysis improves lesion detection; however, ambiguity remains in the low to intermediate AI score range (10–35). Additionally, when radiologists encounter discrepancies between AI predictions and visual assessments, some lesions with low visual suspicion can receive moderately high AI scores, raising uncertainty about whether additional testing is warranted. Conversely, lesions that appear concerning to radiologists may receive unexpectedly low AI scores, complicating clinical decision-making. These discrepancies underscore the importance of identifying optimal thresholds that maximize sensitivity while maintaining specificity [27].

Prior studies have suggested various threshold values, but most have focused on scores ≥ 30, leaving uncertainty about how to interpret low to intermediate AI scores [18,19,20]. Our study proposes that stratifying lesions into two distinct BI-RADS groups with separate AI thresholds may be more clinically effective. This aligns with the broader trend of using AI as a decision-support tool rather than a standalone diagnostic system. Although the potential of AI as a standalone triage tool has been investigated in many studies, the current consensus primarily supports its role as a decision-support system—either augmenting radiologists’ assessments or serving as an alternative to one reader in double-reading workflows [21,28].

Due to the lack of a clear consensus on AI scores, decisions on additional testing often depend on the individual radiologist’s interpretation. This variability can lead to unnecessary additional testing in some cases while potentially overlooking malignancies in others [29].

The selection of optimal AI thresholds depends on the clinical trade-off between sensitivity and specificity. Our ROC analysis demonstrated that AI performance varied by BI-RADS category, with AUC values of 0.69 for BI-RADS 4A and 0.91 for BI-RADS 4B/C, confirming that AI software scoring is more reliable for BI-RADS 4B/C lesions. The overall AUC of 0.87 indicates strong predictive performance, which is comparable to prior studies that have evaluated AI-based mammography screening.

Fourteen previous studies on AI-driven mammography provided AUC values on AI only and combined models. The median AUC performance of the AI image-only models was 0.72 (range 0.62–0.90), compared to 0.61 for breast density or clinical risk factor-based tools (range 0.54–0.69) [30]. Another study by Pertuz et al. showed that the detection performance of the AI systems ranged from low to moderate (AUCs from 0.525 to 0.694), suggesting that our study demonstrated comparable or slightly stronger performance than prior studies [31].

Moreover, consistent with previous research, the AI system demonstrated good performance despite the high proportion (79.9%) of subjects with dense breasts in our study population [16,17].

Our study showed that the AUC (0.69) was lower in the BI-RADS 4A group, which suggests limited discrimination ability in this group, thereby reinforcing the need for cautious interpretation and radiologist oversight. Given that specificity tends to be lower in the 4A category, it is important to note that the AI score alone should not be the sole determinant for assessment.

For BI-RADS 4A lesions, our results suggest that using a lower AI threshold, such as eight (sensitivity = 0.82, specificity = 0.37), ensures that potential malignancies are not overlooked. While increasing the threshold to 10 slightly improves specificity (0.42) at the cost of reduced sensitivity (0.76), maintaining a higher sensitivity is crucial in these 4A cases to minimize missed malignancies [32]. Although a threshold of 19 (sensitivity = 0.71, specificity = 0.64) appears to offer a more balanced trade-off, it still falls short of achieving a specificity above 0.70, making it less suitable for standalone use. Instead, in this group, AI could be more effectively utilized as a secondary tool, applying a lower threshold (e.g., 8 or 10) to enhance lesion detection while leaving the final discrimination of suspicious findings to radiologists. This approach would optimize the strengths of both AI and human expertise, improving overall diagnostic accuracy.

For BI-RADS 4B/4C cases, which exhibit stronger predictive performance (AUC = 0.91), a threshold of 58 may be slightly more favorable in clinical practice, as it achieves a higher sensitivity of 0.85 while maintaining a comparable specificity of 0.85. This balance enables effective cancer detection without substantially increasing false positive rates. Although the Youden-optimal threshold was 63 (sensitivity = 0.82, specificity = 0.92), which also represents an excellent trade-off, the threshold of 58 may be preferred when a modest gain in sensitivity is clinically desirable. These findings support the use of AI scores as a strong independent predictor of malignancy in BI-RADS 4B/4C cases, potentially streamlining biopsy decision-making in this subgroup.

In the overall dataset, a threshold of 35 may be more appropriate for clinical use, as it achieves a higher sensitivity of 0.80 while maintaining a reasonable specificity of 0.78. Although the Youden-optimal threshold was 63 (sensitivity = 0.72, specificity = 0.95), offering excellent specificity and overall balance, the threshold of 35 may be favored in practice due to its better alignment with the goals of early cancer detection, especially considering that breast mammography is a screening tool that is applied to a low-prevalence population, in which false negatives are of particular concern [33].

These findings are consistent with prior studies, in which AI-based mammography models achieved sensitivities between 72.8 and 75% and specificities between 88.3 and 90.8% at different thresholds (Dahlblom et al., 2023; Kizildag Yirgin et al., 2022) [18,19]. Recently, Seker et al. (2024), using the same Lunit AI system, achieved an AUC of 89.6% (86.1–93.2%, 95% CI), and their optimal threshold was 30.44, yielding 72.38% sensitivity and 92.86% specificity [20].

However, our study underscores the risk of using a single threshold across BI-RADS categories, as it led to a significant decline in sensitivity within the BI-RADS 4A group. At a threshold of 35, the sensitivity for BI-RADS 4A cases was only 41.2%, with a specificity of 84.7%. In contrast, BI-RADS 4B/C cases maintained a much higher sensitivity of 88.7%, but with a specificity of 70.2%. This substantial reduction in sensitivity for the 4A group highlights the challenge of applying a uniform threshold across diverse risk categories, as higher cutoffs disproportionately affect malignancy detection in lower-risk lesions, thereby increasing the risk of missed cancers.

These findings suggest that separate thresholds for different BI-RADS categories are necessary to avoid missing cancers in the BI-RADS 4A group. While using a single threshold may reduce workload in general screening, applying category-specific thresholds is preferable for clinical settings, particularly in BI-RADS 4A cases in which malignancy is still possible, even at low AI scores [14].

### 4.2. Clinical Utility and Triage Implications

Although an AI score of ≥35 can be considered a meaningful threshold in general clinical settings, our findings suggest that lower scores—particularly in BI-RADS 4A lesions—require more nuanced interpretation. Due to the inherently ambiguous imaging features of many 4A lesions, such as grouped punctate or amorphous microcalcifications, the AI software may assign relatively low scores, even when malignancy is present. Therefore, radiologists should avoid dismissing regions of interest solely based on low AI scores. If an area flagged by AI, even with a score in the range of 8–10, corresponds to a lesion that could reasonably be classified as BI-RADS 4A upon radiologic assessment, it warrants careful re-evaluation. While a score below 35 might not typically trigger concern, concordance between a low AI score and a radiologically suspicious finding—especially in high-risk patients—should prompt additional scrutiny. In this context, applying a slightly lower AI threshold for 4A lesions (e.g., ≥8–10 or ≥19) may improve sensitivity, supporting the radiologist’s role in integrating AI input within the broader clinical and imaging context.

Furthermore, for lesions categorized as BI-RADS 4B or 4C, an AI score of ≥63 (sensitivity 0.82, specificity 0.92) may be sufficient to support a biopsy recommendation with confidence.

However, increasing sensitivity inevitably leads to a rise in false positives, necessitating efforts to mitigate unnecessary testing. To refine this process, additional investigations were conducted to determine the distinguishing characteristics of discordant cases, such as in the BI-RAD 4A group, with moderate to high AI scores, and in the BI-RAD 4B/4C group, with low AI scores, and compared them with the overall group.

### 4.3. Discordance Patterns in BI-RADS Subgroups

**Figure 4:**
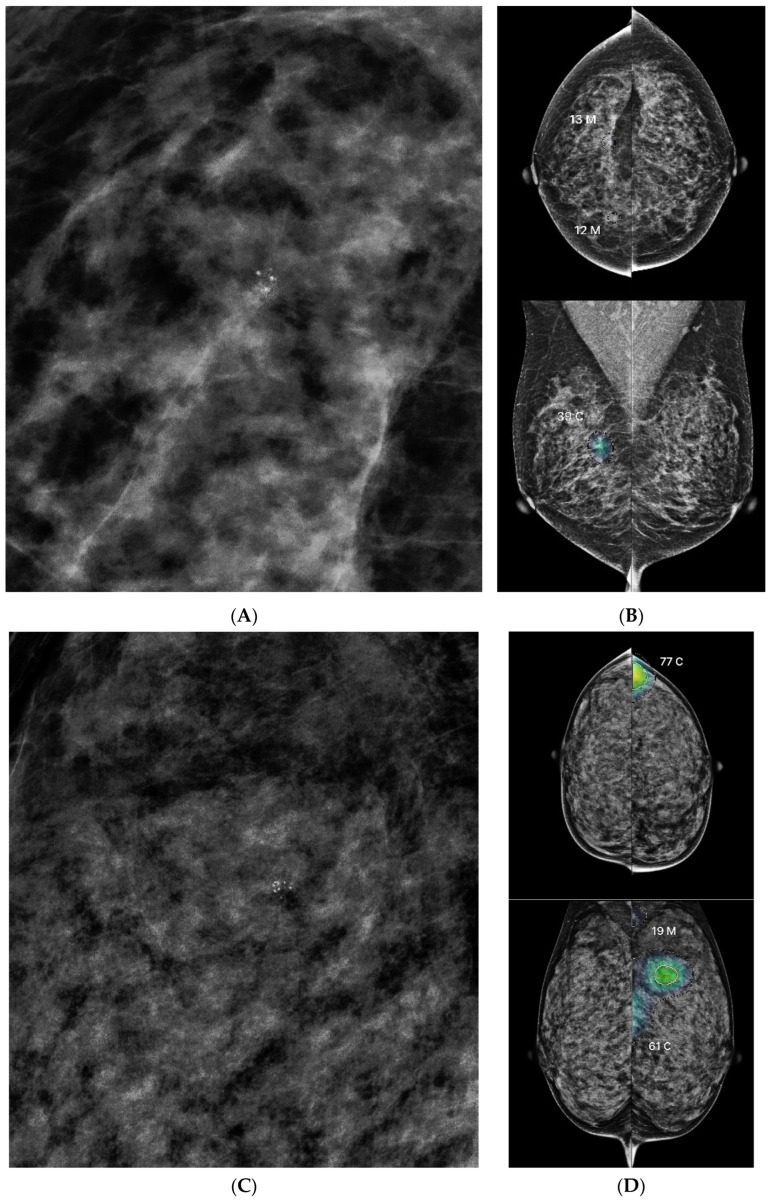
Comparison of two BI-RADS 4A cases with similar mammographic appearances but differing AI scores and pathology. (**A**): Screening mammography of a patient showing right craniocaudal view (magnified) with grouped punctate microcalcifications in the upper outer quadrant. (**B**): AI output demonstrates an AI abnormality score of 39, with the region of interest visualized in a moderately bright color, reflecting a lower estimated malignancy risk. Pathology revealed fibrocystic change. (**C**): Screening mammography of another patient showing left mediolateral oblique view (magnified) with grouped punctate microcalcifications in the upper outer quadrant. (**D**): AI output demonstrates a markedly higher AI abnormality score of 77, with the region of interest appearing significantly brighter, indicating a higher suspicion of malignancy. Pathology confirmed ductal carcinoma in situ (DCIS). Despite similar mammographic appearances and BI-RADS classification, the divergent AI scores correlated with differing pathology results. This comparison underscores the potential of AI scoring to support malignancy risk stratification in ambiguous BI-RADS 4A lesions.

In BI-RADS 4A cases, lesions with moderate to high AI scores (≥35) were predominantly associated with grouped punctate (56.3%) or grouped punctate/amorphous microcalcifications (25%), which often straddle the BI-RADS 3 and 4A boundaries [15]. The markedly higher mean AI scores in malignant cases (73.9, range 38–95) compared to benign cases (45.2, range 35–78) indicate that AI scoring may serve as an independent predictor, assisting radiologists in distinguishing between benign and malignant lesions, particularly when confronted with ambiguous grouped microcalcifications (Figure 4).

However, when all BI-RADS 4A cases with grouped punctate or grouped punctate/amorphous calcifications were analyzed, substantial overlap in AI scores between benign and malignant lesions was observed. Given the small sample size and overlapping distributions, this study does not support a definitive AI threshold or guideline for clinical decision-making in this subgroup. Nevertheless, because grouped microcalcifications constituted the vast majority of BI-RADS 4A cases in our study (*n* = 65, 85.5%), further investigation of this specific lesion group may be clinically valuable. Establishing more refined threshold-based guidance for such cases could improve diagnostic clarity and assist radiologists in navigating ambiguous mammographic findings more confidently.

**Figure 5:**
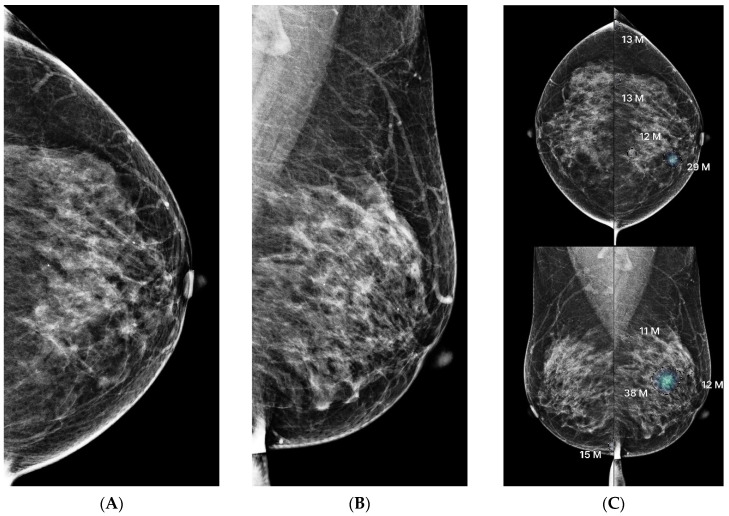
Screening mammography showing a focal asymmetry with moderate AI score later confirmed as malignancy. (**A**,**B**): Left craniocaudal oblique (**A**) and left mediolateral (**B**) views reveal a 1 cm focal asymmetry in the upper inner quadrant, initially interpreted as BI-RADS 4A. (**C**): Output from the AI system shows the lesion highlighted in the region of interest color map, with an AI abnormality score of 38. Although this AI score was within the moderate range, it exceeded the proposed sensitivity-prioritized threshold (≥35). Ultrasound-guided core biopsy later confirmed invasive ductal carcinoma with spiculated margins.

Discordant BI-RADS 4A cases with AI scores of ≥35 demonstrated a malignancy rate of 43.8%, nearly double that of the overall 4A group (22.4%). This significant difference indicates that AI scores in the moderate-to-high range, even within a lower-risk BI-RADS category, may carry meaningful diagnostic value. Such findings support the use of AI scoring as a supplementary triage tool, encouraging closer scrutiny or earlier follow-up in otherwise ambiguous cases. One such case in the BI-RADS 4A group, with a moderate to high AI score, exemplifies this observation: a 1 cm focal asymmetry with an AI score of 38 was initially classified as BI-RADS 4A but was later retrospectively confirmed as malignant, exhibiting spiculated margins. Although the AI score was not extremely high, it exceeded the 35-point threshold, highlighting the need for careful reassessment, even when AI scores fall within the moderate range (Figure 5).

Conversely, lesions with lower AI scores (<35) in BI-RADS 4B/C cases commonly exhibited segmental distribution microcalcifications, which are traditionally considered suspicious. However, despite their concerning morphologies, the malignancy rate in these low-score cases was significantly lower (11.8%) than that of the overall 4B/C group (60.2%), suggesting that the AI scores effectively down-triage cases with suspicious characteristics [14].

Further analysis of lesion morphology revealed that amorphous and amorphous/punctate microcalcifications accounted for 94.7% of cases in the low-score group, distinguishing them from the broader 4B/C cohort, which exhibited a more diverse range of suspicious microcalcifications. This suggests that the AI system tends to assign lower scores to amorphous calcifications, likely due to their ambiguous classification within BI-RADS 3 and 4 [15].

Moreover, in these low-score cases, there was no significant difference in the AI scores between malignant and benign lesions. This suggests that once the AI system assigns a low score, it becomes challenging to differentiate between malignancy and benignity based solely on the AI score. However, when considering low-score cases in the 4B/C group, the malignancy rate was markedly lower than that of the overall 4B/C group (60.2% vs. 19.5%), indicating that the AI system does contribute to filtering out a significant proportion of non-malignant cases [14].

### 4.4. Limitations and Future Directions

Despite its strong findings, this study has limitations. First, it was conducted at a single center with a single radiologist, which may limit its generalizability. Expanding the study to multi-center datasets with diverse radiologist interpretations would improve AI validation. Second, the retrospective nature of the study excluded follow-up cases that did not undergo biopsy, leading to the potential overestimation of malignancy rates, particularly in BI-RADS 4A cases. Third, AI model performance may shift over time due to software updates, necessitating continued monitoring to ensure reliability. Fourth, the dataset size was relatively limited due to the restricted number of eligible cases during the study period. While the current sample provided meaningful insights into threshold behavior within BI-RADS 4 lesions, a larger dataset with more diverse patient demographics would enhance the statistical robustness and generalizability of the findings. Future studies should aim to increase sample size through extended data collection periods and multi-center collaboration.

Furthermore, future research should explore the prospective validation of AI-based threshold recommendations, incorporating long-term clinical outcomes to refine AI decision-making in mammography. Additionally, developing hybrid AI–human interpretative models, where AI acts as an intelligent triage system, may further enhance clinical efficiency while preserving diagnostic accuracy.

## 5. Conclusions

This study offers both theoretical and clinical contributions to the use of AI in mammographic evaluations. Theoretically, it proposes a BI-RADS-specific threshold optimization approach that moves beyond fixed-score systems, aligning AI interpretation more closely with radiologic classification standards. Clinically, this stratified thresholding has the potential to assist radiologists—particularly in borderline BI-RADS 4A cases—by providing additional risk context that may reduce unnecessary biopsies while preserving diagnostic safety.

Although several threshold strategies were proposed for BI-RADS 4A lesions based on score distributions, overlap between benign and malignant cases remains a challenge, and the thresholds should be applied with caution, as current AI systems may not be reliable enough for independent decision-making in the absence of radiologist input.

Radiologist oversight remains essential, and future studies should aim to refine threshold strategies in specific lesion subtypes to support more nuanced AI-assisted decision-making in breast cancer screening.

## Figures and Tables

**Table 1:** Summary of prior studies on AI thresholds in mammography.

Study	AI Tool	Threshold	Sensitivity	Specificity	BI-RADS-Specific?
Dahlblom et al. (2021) [18]	Not stated	62	75%	90.8%	No
Kizildag Yirgin et al. (2022) [19]	Commercial AI	34.5	72.8%	88.3%	No
Seker et al. (2024) [20]	Lunit	30.4	72.4%	92.9%	No

**Table 2:** Comparison of mean AI abnormality scores according to breast density, lesion features, and clinical outcomes in 194 subjects.

Category	*n* (%)	AI Score (Mean ± SD)	*p*-Value
BIRADS Category			<0.001
BI-RADS A	76 (39.2%)	22.38 ± 22.74	
BI-RADS B/C	118 (60.8%)	58.14 ± 37.93	
Breast Density			0.003
Fatty breast (Density 1 and 2)	39 (20.1%)	61.90 ± 36.02	
Dense breast (Density 3 and 4)	155 (79.9%)	39.66 ± 36.18	
Mass Margins			<0.001
Circumscribed	14 (7.2%)	46.29 ± 38.18	
Irregular	33 (17.0%)	62.67 ± 36.78	
Spiculated	17 (8.8%)	86.47 ± 23.91	
Mass Density			0.002
Iso-dense	24 (12.4%)	51.62 ± 37.99	
Hyper-dense	40 (20.6%)	73.67 ± 33.51	
Architectural Distortion	16 (8.2%)	79.94 ± 31.47	<0.001
Microcalcifications-Distribution			0.012
Grouped	87 (44.8%)	34.24 ± 32.09	
Segmental	69 (35.6%)	45.75 ± 39.10	
Microcalcifications-Morphology			<0.001
Punctate	48 (24.7%)	21.81 ± 22.08	
Amorphous	24 (12.4%)	35.25 ± 33.35	
Punctate/Amorphous	58 (29.9%)	33.59 ± 31.79	
Coarse Heterogeneous	8 (4.1%)	78.25 ± 21.79	
Fine Pleomorphic	15 (7.7%)	97.60 ± 2.44	
Fine Linear/Branching	3 (1.5%)	68.33 ± 52.25	
Biopsy Results			<0.001
Benign Lesions	106 (54.6%)	20.91 ± 20.89	
Malignant Lesions	88 (45.4%)	72.11 ± 32.67	
Recommendations			<0.001
Follow-Up (FU)	43 (22.2%)	18.93 ± 18.13	
Biopsy or US (Recall)	151 (77.8%)	51.31 ± 38.07	
Ultrasound Detectability			<0.001
Detected on US	87 (44.8%)	66.36 ± 30.97	
Not detected on US	107 (55.2%)	32.97 ± 28.06	

Statistical significance was evaluated using Welch’s *t*-tests (BIRADS category), independent *t*-tests (breast density, mass density, architectural distortion, microcalcification distribution, biopsy results, recommendations, and ultrasound detectability), and one-way ANOVA (mass margins and microcalcification morphology). Statistically significant *p*-values (<0.05) are shown, highlighting associations between higher AI scores and features such as spiculated margins, architectural distortion, segmental microcalcifications (distribution), coarse heterogeneous, fine pleomorphic, and fine linear/branching microcalcifications (morphology), as well as malignancy. AI = artificial intelligence, US = ultrasound.

**Table 3:** Threshold comparison for AI-based malignancy prediction across BI-RADS subgroups.

Group	Threshold Type	Threshold	Sensitivity	Specificity	Youden Index
4A	Youden-optimal	19	0.706	0.644	0.350
4A	Sensitivity-prioritized	10	0.765	0.424	0.189
4A	Sensitivity-prioritized	8	0.824	0.373	0.197
4B/4C	Youden-optimal	63	0.817	0.915	0.732
4B/4C	Sensitivity-prioritized	58	0.845	0.851	0.696
Overall	Youden-optimal	63	0.716	0.953	0.669
Overall	Sensitivity-prioritized	35	0.795	0.783	0.578

This table summarizes threshold selection strategies for AI abnormality scores in the BI-RADS 4A, 4B/4C, and overall groups. Thresholds were determined using Youden’s index to balance sensitivity and specificity, as well as sensitivity-prioritized alternatives to minimize the risk of missed cancers. The results highlight trade-offs between specificity and sensitivity, supporting tailored threshold selection based on clinical context. AUC values and resulting trade-offs between sensitivity and specificity are shown to guide clinical threshold selection.

## Data Availability

The data are not publicly available due to institutional policy and patient privacy restrictions. Data may be available upon reasonable request and subject to institutional approval.

## Abbreviations

The following abbreviations are used in this manuscript:

AI	Artificial Intelligence
AUC	Area Under the Curve
BI-RADS	Breast Imaging Reporting and Data System
CADe/x	Computer-Aided Detection/Diagnosis
CC	Craniocaudal (Mammographic View)
CI	Confidence Interval
DCIS	Ductal Carcinoma In Situ
FDA	Food and Drug Administration
IDC	Invasive Ductal Carcinoma
IRB	Institutional Review Board
KoNIBP	Korea National Institute for Bioethics Policy
LCIS	Lobular Carcinoma In Situ
MFDS	Ministry of Food and Drug Safety
MLO	Mediolateral Oblique (Mammographic View)
ROC	Receiver Operating Characteristic
SD	Standard Deviation
US	Ultrasound

## References

[1] Kim J., Harper A., McCormack V., Sung H., Houssami N., Morgan E., Mutebi M., Garvey G., Soerjomataram I., Fidler-Benaoudia M.M. (2025). Global Patterns and Trends in Breast Cancer Incidence and Mortality across 185 Countries. Nat. Med..

[2] Anderson B.O., Braun S., Lim S., Smith R.A., Taplin S., Thomas D.B. (2003). Global Summit Early Detection Panel Early Detection of Breast Cancer in Countries with Limited Resources. Breast J..

[3] Marmot M.G., Altman D.G., Cameron D.A., Dewar J.A., Thompson S.G., Wilcox M. (2013). The Benefits and Harms of Breast Cancer Screening: An Independent Review. Br. J. Cancer.

[4] Lehman C.D., Wellman R.D., Buist D.S.M., Kerlikowske K., Tosteson A.N.A., Miglioretti D.L. (2015). Breast Cancer Surveillance Consortium Diagnostic Accuracy of Digital Screening Mammography with and Without Computer-Aided Detection. JAMA Intern. Med..

[5] Schaffter T., Buist D.S.M., Lee C.I., Nikulin Y., Ribli D., Guan Y., Lotter W., Jie Z., Du H., Wang S. (2020). Evaluation of Combined Artificial Intelligence and Radiologist Assessment to Interpret Screening Mammograms. JAMA Netw. Open.

[6] Kontos D., Conant E.F. (2019). Can AI Help Make Screening Mammography “Lean”?. Radiology.

[7] Rodríguez-Ruiz A., Krupinski E., Mordang J.-J., Schilling K., Heywang-Köbrunner S.H., Sechopoulos I., Mann R.M. (2019). Detection of Breast Cancer with Mammography: Effect of an Artificial Intelligence Support System. Radiology.

[8] Kim H.-E., Kim H.H., Han B.-K., Kim K.H., Han K., Nam H., Lee E.H., Kim E.-K. (2020). Changes in Cancer Detection and False-Positive Recall in Mammography Using Artificial Intelligence: A Retrospective, Multireader Study. Lancet Digit. Health.

[9] Lee J.H., Kim K.H., Lee E.H., Ahn J.S., Ryu J.K., Park Y.M., Shin G.W., Kim Y.J., Choi H.Y. (2022). Improving the Performance of Radiologists Using Artificial Intelligence-Based Detection Support Software for Mammography: A Multi-Reader Study. Korean J. Radiol..

[10] McKinney S.M., Sieniek M., Godbole V., Godwin J., Antropova N., Ashrafian H., Back T., Chesus M., Corrado G.S., Darzi A. (2020). International Evaluation of an AI System for Breast Cancer Screening. Nature.

[11] Conant E.F., Toledano A.Y., Periaswamy S., Fotin S.V., Go J., Boatsman J.E., Hoffmeister J.W. (2019). Improving Accuracy and Efficiency with Concurrent Use of Artificial Intelligence for Digital Breast Tomosynthesis. Radiol. Artif. Intell..

[12] Larsen M., Aglen C.F., Lee C.I., Hoff S.R., Lund-Hanssen H., Lång K., Nygård J.F., Ursin G., Hofvind S. (2022). Artificial Intelligence Evaluation of 122 969 Mammography Examinations from a Population-Based Screening Program. Radiology.

[13] Lee S.E., Hong H., Kim E.-K. (2024). Positive Predictive Values of Abnormality Scores From a Commercial Artificial Intelligence-Based Computer-Aided Diagnosis for Mammography. Korean J. Radiol..

[14] Dembrower K., Wåhlin E., Liu Y., Salim M., Smith K., Lindholm P., Eklund M., Strand F. (2020). Effect of Artificial Intelligence-Based Triaging of Breast Cancer Screening Mammograms on Cancer Detection and Radiologist Workload: A Retrospective Simulation Study. Lancet Digit. Health.

[15] American College of Radiology Breast Imaging Reporting and Data System (BI-RADS). ACR.org. https://www.acr.org/Clinical-Resources/Clinical-Tools-and-Reference/Reporting-and-Data-Systems/BI-RADS.

[16] Koch H.W., Larsen M., Bartsch H., Kurz K.D., Hofvind S. (2023). Artificial Intelligence in BreastScreen Norway: A Retrospective Analysis of a Cancer-Enriched Sample Including 1254 Breast Cancer Cases. Eur. Radiol..

[17] Kwon M.-R., Chang Y., Ham S.-Y., Cho Y., Kim E.Y., Kang J., Park E.K., Kim K.H., Kim M., Kim T.S. (2024). Screening Mammography Performance According to Breast Density: A Comparison between Radiologists versus Standalone Intelligence Detection. Breast Cancer Res. BCR.

[18] Dahlblom V., Andersson I., Lång K., Tingberg A., Zackrisson S., Dustler M. (2021). Artificial Intelligence Detection of Missed Cancers at Digital Mammography That Were Detected at Digital Breast Tomosynthesis. Radiol. Artif. Intell..

[19] Kizildag Yirgin I., Koyluoglu Y.O., Seker M.E., Ozkan Gurdal S., Ozaydin A.N., Ozcinar B., Cabioğlu N., Ozmen V., Aribal E. (2022). Diagnostic Performance of AI for Cancers Registered in A Mammography Screening Program: A Retrospective Analysis. Technol. Cancer Res. Treat..

[20] Seker M.E., Koyluoglu Y.O., Ozaydin A.N., Gurdal S.O., Ozcinar B., Cabioglu N., Ozmen V., Aribal E. (2024). Diagnostic Capabilities of Artificial Intelligence as an Additional Reader in a Breast Cancer Screening Program. Eur. Radiol..

[21] Chang Y.-W., An J.K., Choi N., Ko K.H., Kim K.H., Han K., Ryu J.K. (2022). Artificial Intelligence for Breast Cancer Screening in Mammography (AI-STREAM): A Prospective Multicenter Study Design in Korea Using AI-Based CADe/x. J. Breast Cancer.

[22] DeLong E.R., DeLong D.M., Clarke-Pearson D.L. (1988). Comparing the Areas under Two or More Correlated Receiver Operating Characteristic Curves: A Nonparametric Approach. Biometrics.

[23] Youden W.J. (1950). Index for Rating Diagnostic Tests. Cancer.

[24] Zhao T., Fu C., Song W., Sham C.-W. (2024). RGGC-UNet: Accurate Deep Learning Framework for Signet Ring Cell Semantic Segmentation in Pathological Images. Bioengineering.

[25] Zhao T., Fu C., Tian Y., Song W., Sham C.-W. (2023). GSN-HVNET: A Lightweight, Multi-Task Deep Learning Framework for Nuclei Segmentation and Classification. Bioengineering.

[26] Gu Y., Fu C., Song W., Wang X., Chen J. (2025). RTLinearFormer: Semantic Segmentation with Lightweight Linear Attentions. Neurocomputing.

[27] Smith M., Heath Jeffery R.C. (2020). Addressing the Challenges of Artificial Intelligence in Medicine. Intern. Med. J..

[28] Dembrower K., Crippa A., Colón E., Eklund M., Strand F. (2023). ScreenTrustCAD Trial Consortium Artificial Intelligence for Breast Cancer Detection in Screening Mammography in Sweden: A Prospective, Population-Based, Paired-Reader, Non-Inferiority Study. Lancet Digit. Health.

[29] Zeng A., Houssami N., Noguchi N., Nickel B., Marinovich M.L. (2024). Frequency and Characteristics of Errors by Artificial Intelligence (AI) in Reading Screening Mammography: A Systematic Review. Breast Cancer Res. Treat..

[30] Schopf C.M., Ramwala O.A., Lowry K.P., Hofvind S., Marinovich M.L., Houssami N., Elmore J.G., Dontchos B.N., Lee J.M., Lee C.I. (2024). Artificial Intelligence-Driven Mammography-Based Future Breast Cancer Risk Prediction: A Systematic Review. J. Am. Coll. Radiol. JACR.

[31] Pertuz S., Ortega D., Suarez É., Cancino W., Africano G., Rinta-Kiikka I., Arponen O., Paris S., Lozano A. (2023). Saliency of Breast Lesions in Breast Cancer Detection Using Artificial Intelligence. Sci. Rep..

[32] Hayashida T., Odani E., Kikuchi M., Nagayama A., Seki T., Takahashi M., Futatsugi N., Matsumoto A., Murata T., Watanuki R. (2022). Establishment of a Deep-Learning System to Diagnose BI-RADS4a or Higher Using Breast Ultrasound for Clinical Application. Cancer Sci..

[33] Evans K.K., Birdwell R.L., Wolfe J.M. (2013). If You Don’t Find It Often, You Often Don’t Find It: Why Some Cancers Are Missed in Breast Cancer Screening. PLoS ONE.

